# What Do Turkish Parents Think About Using Bee Products for Their Children?

**DOI:** 10.3390/foods14203532

**Published:** 2025-10-16

**Authors:** Selin İkiz, Merve Keskin, Figen Gürsoy

**Affiliations:** 1 Vocational School of Health Services, Bilecik Şeyh Edebali University, Bilecik 11210, Türkiye; merveozdemirkeskin@gmail.com; 2 Department of Child Development, Faculty of Health Science, Ankara University, Ankara 06100, Türkiye; fgursoy@ankara.edu.tr

**Keywords:** child nutrition, child development, bee products, parent, immune system

## Abstract

Healthy nutrition is an essential factor in the growth and development of children. To support children’s developmental processes and protect them from diseases, their immune systems must be strengthened through a balanced diet. Bee products are natural substances with high nutritional value. Although various studies show the benefits of bee products on human health, there are uncertainties among parents regarding their use. Therefore, the study aimed to investigate parents’ awareness of using bee products for their children and to determine their perceptions about the effects of these products on their health. This qualitative study employed semi-structured interviews with 40 parents of children aged 4–6 years. It was found that the bee products consumed by their children were primarily honey, with some parents also using propolis and pollen. In cases where children did not want to consume bee products, parents mixed bee products with other foods to make them more palatable. Parents use natural products to support their children’s development and protect them from diseases, and generally prefer honey as a bee product.

## 1. Introduction

Developmental processes in early childhood have a profound impact on lifelong health and well-being. Proper nutrition is crucial for children experiencing rapid growth from an early age [1]. It is essential for both development and disease resistance [2]. Inadequate and unbalanced nutrition during childhood can delay growth, alter body measurements, weaken immunity, increase disease risk, and even lead to death [3]. Therefore, boosting children’s immune systems from an early age through proper nutrition is vital for safeguarding their health and promoting their growth.

Consumers mainly know about honey, and they know less about propolis, royal jelly, and pollen. The studies indicate that consumers would like to know more about bee products. They also would not like to purchase fake bee products [4,5]. Thus, the bee products which were produced according to certified ethical and sustainable beekeeping practices were more popular for the treatment of illnesses such as cardiovascular diseases, bacterial infections, dental diseases, and more [6,7]. Parents can also use natural foods to strengthen children’s immunity and aid recovery from illness [8]. They may turn to natural products due to potential side effects and antibiotic resistance. Common choices are bee products, such as honey, pollen, and propolis. All bee products are rich in different biocompounds such as antioxidants, organic acids, proteins, phenolic compounds, and flavonoids [9]. Their regular consumption can help children resist infections [10]. During colds and flu, honey soothes the throat and eases breathing [11]. Its prebiotic properties also support digestive health [10]. However, honey may contain *Clostridium botulinum* spores, which can cause infant botulism; therefore, it should not be given to infants under one year of age [10]. Thus, proper use of honey according to age is important. Propolis, another bee product, possesses antimicrobial, antiviral, and antifungal properties and may help boost children’s immune systems and help treat upper respiratory tract infections and other diseases [12,13,14]. Lesser-known bee products, such as bee pollen, bee bread (perga), and royal jelly, can also serve as a dietary supplement with varied formulations [15,16]. While bee products are widely used by adults, they should be administered cautiously, especially in children with allergies [17,18].

Young children rely on their parents for nutrition, making parental attitudes and opinions crucial [19]. Although the available literature data, obtained mainly in laboratory conditions, show the role of bee products in pediatric nutrition and diseases, there are limited studies on parents’ views about using bee products for their children. This study, therefore, aims to investigate parents’ awareness and perceptions of the use of bee products on children’s health.

## 2. Method

### 2.1. Research Design

Phenomenology, a qualitative research approach, was used in the study to examine parents’ opinions on using bee products for their children, explore their thoughts on the health effects of these products in detail, understand their perceptions and experiences, and gather rich data sources. The goal of phenomenological research is to explore how individuals who experience the target phenomenon interpret their experiences from their own perspectives and to identify common themes in these experiences [20].

### 2.2. Study Group

The criterion sampling method, a type of purposeful sampling, was used to determine the study group for this study. In the criterion sampling method, the researcher must determine specific criteria suitable for the study. Participants fulfilling these criteria are included in the study [21]. Although the relevant sampling method allows obtaining in-depth information from participants who meet the criteria, the findings cannot be generalized to large masses due to the non-representativeness of the sample. In this direction, parents who met the criteria for volunteering to participate in the study, had a child/children aged 4–6 years, and lived in the city center in Türkiye were included in the study group. The saturation point of the data obtained during the interviews was considered when determining the exact number of participants to include in the study group. When researchers encounter repetitions in interviews, they should realize that the data has reached a saturation point [22]. For this reason, the study was conducted with 40 parents, as the interview data were repetitive and redundant. A descriptive list of participating parents is given in Table 1.

### 2.3. Data Collection Tools

In the study, a Demographic Information Form was prepared to determine the demographic characteristics (age, education level, number of children, etc.) of the study group. Additionally, a semi-structured interview form was prepared based on the relevant literature to explore parents’ use of bee products for their children and their thoughts on these products. The questions in the semi-structured interview form were submitted to the opinions of three field experts regarding their suitability for the research purpose, clarity, and comprehensibility. Adjustments were made in line with the feedback received, and then pilot interviews were conducted with three parents. In the pilot interviews, the comprehensibility of the questions was evaluated, and the interview form was finalized. The final version of the questions is listed below.

(1) What do you do to protect your child or children from diseases?
(2) Do you consume bee products for your child or children? If yes, which bee products do you prefer?
(3) Which bee product do you use and for what purpose or purposes?
(4) What do you pay attention to when choosing bee products for your child or children?
(5) Do you face any difficulties in getting your child or children to consume bee products? If yes, what are the difficulties you face? How do you overcome these difficulties?
(6) Where do you obtain bee products?

### 2.4. Data Collection Procedure

Ethics Committee authorization was obtained prior to commencing the data collection process (Ethical Approval no: E-54674167-050.04-296011). The purpose of the study was then explained to the potential participants. It was stated that participation in the study was entirely voluntary, that the answers provided to the questions would be used solely within the scope of the scientific study in accordance with the principles of confidentiality, and that participants could withdraw from the study at any stage of the research process. Following this, written consent was obtained from the participants, who voluntarily agreed to participate in the study. In the interviews, parents were asked for permission to be audio-recorded. The interviews of the parents who gave permission were audio-recorded for nearly 25 min. The interviews were planned in advance to ensure comfortable communication with the parents and were conducted in a suitable environment (office, home, etc.) where the researcher and the participant could easily see each other and where there was no noise and interruptions. One-to-one in-depth interviews were conducted by the researcher without the use of observers and reporters. The answers given by the parents who did not permit the questions during the interview were transcribed by the researcher.

### 2.5. Data Analysis

Content analysis was employed to analyze the data collected from the interviews with the study group. In content analysis, data that are similar to each other are classified, organized, interpreted, and evaluated within the framework of specific codes and themes [23]. Accordingly, all interviews were transcribed and transferred to the MAXQDA 24 software without any changes. MAXQDA software enables the systematic analysis of data and the use of visual analysis tools [24]. While the data were transferred to the program, the participants were given code numbers between P1 and P40. Then, the participants’ responses were subjected to content analysis by two researchers, and codes and subcodes were created. Additionally, the frequencies of the answers obtained through coding were determined. The researchers first coded independently and then compared their coding to ensure coding consistency. After the comparison, the researchers reached a consensus, and the coding was finalized. While presenting the findings, participant statements were given to exemplify the coding. The code and order of the participants were indicated in brackets at the end of the relevant sentences.

## 3. Results

This study aimed to investigate the use of bee products by parents for their children and to gather their thoughts on the potential health effects of these products. In the research, the measures taken by parents to protect their children from diseases were examined first. Afterward, the data were analyzed to reveal how parents use bee products in relation to their children’s health.

### 3.1. Protecting Their Children from Diseases

**Figure 1:**
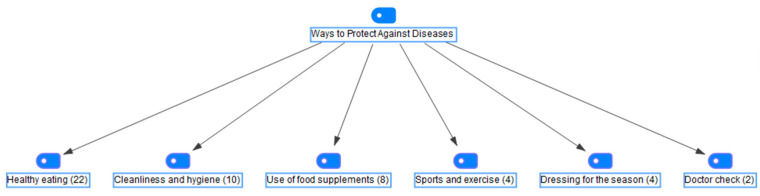
The ways parents can protect their children from diseases.

In the study, semi-structured interview notes were analyzed using MAXQDA software. Figure 1 shows that parents attempt to protect their children from diseases by employing various methods. The prevention methods frequently mentioned by the parents in the interviews were healthy nutrition (44%), cleanliness and hygiene (20%), use of food supplements (16%), sports and exercise (8%), seasonal dressing (8%), and doctor control (4%).

In the interviews, one of the participants stated that they attach importance to healthy eating to strengthen the immunity of their children, with the statement, ‘I pay attention to healthy consumption habits. I ensure that they consume seasonal fruits and vegetables. I need to maintain their high immunity’ (P9). Another parent said, ‘I pay attention to the consumption of fruit and vegetables. Even washing and cleaning them is important. I give probiotics and supplements. In crowded environments, after visiting a park or a garden, I always make them take a shower’ (P25), emphasizing that she tried to protect her children from diseases in many ways. In summary, the results of the interview analyses showed that parents try to protect their children against diseases in many ways, especially by focusing on their children’s healthy nutrition. Due to emotional responsibility, it could be stated that it was compatible with parenting.

### 3.2. Aspects of Parents’ Use of Bee Products for Their Children

**Figure 2:**
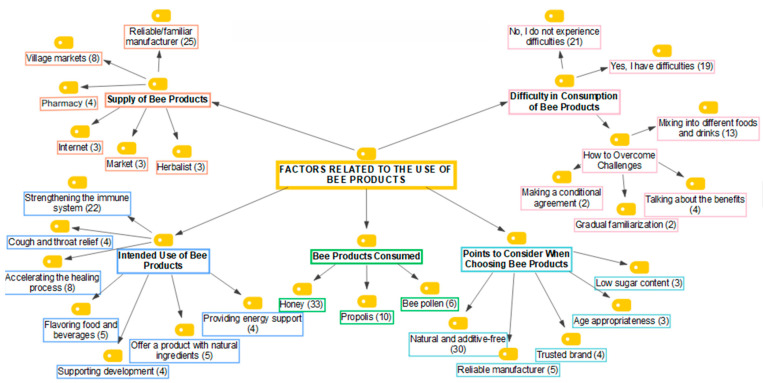
Factors related to parents’ use of bee products for their children.

Parents could use natural foods in the healthy nutrition process to protect their children from diseases. Bee products are among the natural products that can be preferred in the nutrition process. In the study, analyses were conducted to determine parents’ opinions on the use of bee products for their children, and the findings are presented in Figure 2.

Figure 2 illustrates that parents’ opinions on the use of bee products for their children were categorized into five groups. These categories include: consumption of bee products, intended use of bee products, considerations for preferring bee products, difficulties in consuming bee products, and the supply of bee products.

In the category of bee products consumed, it was determined that parents consumed mostly honey (68%), propolis (21%), and pollen (12%) for their children. During the interviews, one of the participants said, ‘My children love honey very much; all my children eat it for breakfast. I also give them pollen, but they do not like it because it tastes bad. That is why I do not give pollen very intensively. I try to give it only when I think they will get sick’ (P26), and stated that honey is more preferred by children because of its sweet taste. Additionally, the participant emphasized that he evaluated these products based on their health benefits. Another participant said, ‘We also have pollen and royal jelly at home. However, children did not prefer them much. We generally consume honey. We prefer the most natural one. We do not use ready-market honey’ (P5) and stated that children prefer honey over other bee products. Another participant said, ‘We usually prefer honey by buying it from people we know. We used to use propolis before, but after a while, one of our doctors told us that propolis has the risk of causing early puberty.’ That is why we no longer use propolis (P30) and have emphasized that they generally prefer honey, but they have concerns about propolis. It could be concluded that honey was more preferable for parents in terms of both taste and accessibility. On the other hand, these findings reveal that “naturalness” and “reliable producer” criteria were the determinants of parents’ orientation towards natural and additive-free products.

In the category of the purposes of use of bee products, the most frequently stated purposes of use by parents were as follows: strengthening the immune system of their children (42%), accelerating the healing process (15%), providing a product with natural ingredients (9.5%), flavoring food and drinks (9.5%), relieving cough and throat (8%), supporting development (8%), and providing energy support (8%). In the interviews, the participants reiterated these codes about the purposes of use of bee products by saying, ‘I give honey both to strengthen the immunity of children, and I use it instead of sugar’ (P16). Another participant emphasized the use of different bee products for health purposes with the statement, ‘I try to give honey and propolis to my child to heal faster when he/she is sick, especially in winter months’ (P28). As a result, it was understood that parents prefer different bee products for various purposes in their children’s care.

In the category of issues considered when choosing bee products, it was determined that parents paid more attention to the bee product being natural and additive-free (67%) when selecting bee products for their children. Apart from this, it was determined that they paid attention to reliable producer (11%), reliable brand (9%), age appropriateness (6.5%), and low sugar content (6.5%). During the interviews, one of the participants said, ‘I pay attention to the fact that it is natural. I examine the label and packaging, paying attention to whether it contains glucose syrup or preservatives’ (P28) and emphasized that it is important for parents that bee products do not contain additives. Another participant emphasized the importance of reliable producers in the preference of bee products, as well as careful and conscious use, with the statement, ‘I pay attention to buy the products that I think are natural from the producers I know and trust. I also try to feed it by researching and checking if the product is suitable for my child’s age’ (P15).

In the category of difficulties experienced with the consumption of bee products, although many parents reported no issues (52.5%), it was observed that some parents faced challenges with their children’s consumption of these products (47.5%). When the methods of overcoming the difficulties experienced by parents were analyzed, it was found that parents most often used the method of mixing bee products into various foods and beverages (62%) for their children who did not want to consume bee products. In addition, it was determined that they also employed methods such as mentioning the benefits of bee products (19%), making conditional agreements (9.5%), and gradual acclimatization (9.5%). In the interviews, one of the participants stated that he solved this problem by mixing it with different products by saying, ‘We mix it with yoghurt because we have difficulty in making them consume it plain’ (P6). Another participant said, ‘My children are not very sweet-loving children. I have difficulty feeding them honey from time to time, but I try to encourage them to eat it by saying things like, ‘It will be perfect for you against diseases,’ ‘you will feel better,’ and ‘eat a spoonful of this.’ Apart from that, we try to encourage them to eat honey by saying things like, ‘If you eat this, I will give you this.’ We do our best to support the physical and mental well-being of our children’ (P5) by highlighting that bee products are consumed through various methods.

In the category of procurement of bee products, it was determined that parents procured bee products mostly from trusted/familiar producers (54%), village markets (17.5%), pharmacies (9%), the internet (6.5%), markets (6.5%), and herbalists (6.5%). During the interviews, one of the participants said, “We usually buy from the producers around us, whom we know and trust. We never buy branded products sold commercially (P30) and prefer to buy from producers we know instead of branded products. Another participant said, ‘We buy honey from producers we know, recognize, and trust. I prefer to buy pollen from the herbalist I trust’ (P27) and emphasized that they procure different bee products from different sellers.

In summary, the results of the interview analyses showed that the bee products consumed by the parents for their children were primarily honey, and they mostly used these products to strengthen their children’s immune systems. Additionally, it was found that parents were more likely to pay attention to the fact that the product was natural and additive-free, while also preferring products made from bee products. In addition, the results of the interview analyses showed that parents who had difficulty consuming bee products offered these products to their children by mixing them into various foods and drinks, and that parents mainly obtained bee products from reliable/familiar producers.

## 4. Discussion

Upon examining the literature, it was observed that studies have been conducted to determine families’ views on the use of food supplements for their children. In a study, it was reported that products from traditional and complementary medicine practices (such as plants and bee products) were used not only by adults but also by children [25]. The data from 20 European countries were analyzed, and it was determined that 56% (range: 10–90%, adjusted for population size) of the European population used complementary and alternative medicine at least one time. Using complementary and alternative medicine for children ranged from 52% (range: 5–90%, adjusted for population size). It was reported that there was an increasing awareness of pediatric complementary and alternative medicine [25]. A study conducted by Marimuthu [26] addresses the antecedents and consequences of herbal food supplement use for children from a consumer perspective. The fourteen young mothers from Generation Y (Gen Y) were chosen as key factors, and 67% questionnaires were obtained. A total of 1007 young mothers who currently use herbal food supplements primarily for disease prevention in their children completed a self-administered, structured questionnaire. Social group influence, perceived benefits, and perceived convenience were found to be the primary factors influencing young mothers’ use of herbal food supplements for their children. The study also emphasized the importance of peer-to-peer influence through social networks in raising awareness and influencing other young mothers to use herbal supplements. In a study conducted by Kang [27], the orientations of Korean families towards food supplements were analyzed between 2014 and 2018. An offline survey was conducted, and 381, 426, 301, 519, and 369 surgeries were obtained, respectively. According to this study, the proportion of participants who perceived “food safety” as the most important factor when purchasing processed food, while viewing “food additives” as the biggest threat to food safety, has decreased over the years. However, most consumers still have negative perceptions of food additives [27]. In this study, it was found that Korean parents dislike food additives, highlighting the need for multifaceted educational programs to communicate scientific knowledge about them. Piekara et al. [28] conducted a study on the views of Polish parents and caregivers regarding the use of nutritional supplements for children aged 3–12 years. A questionnaire was performed on parents to find out their knowledge, practice, and attitudes toward dietary supplements. The study was limited to Polish parents (population of 600,000), and an online survey was performed. It was found that parents who administered nutritional supplements to their children tended to trust such products more than those who did not. It was also confirmed that people who took nutritional supplements passed on their behavior patterns to their children [28]. From this perspective, it was understood that parents may prefer herbal products for their children. These studies also support the results of the present study. The study was limited because the population did not include the entire country. The study group was dominantly female, and other factors, such as the relationship between gender and attitudes or behaviors, were not examined [28]. When the literature was examined, studies on the effects of various bee products, conducted mainly in laboratory conditions, were found to impact child health [29,30,31]. In general, it was stated that bee products could be used in the treatment of upper respiratory tract infections or other microorganism-related diseases in children. However, it was also emphasized that bee products may be allergenic and should not be used without allergy tests [18,31,32,33].

There were different studies conducted with different limitations and ways in the literature, but there was a limited study determining parents’ opinions about bee products. In a study conducted by Živanović et al. [34], attitudes and prevalence of using bee products in pediatric pulmonology patients were examined. A cross-sectional study was conducted in the pediatric pulmonology outpatient clinic using a questionnaire with 20 open-ended and closed questions. According to this study, 120 of the 138 questionnaires distributed were fully completed and included in the analysis. 79% of respondents gave their children some form of bee product to alleviate health problems (e.g., asthma and bronchitis), most commonly meadow honey, propolis, and royal jelly. These studies demonstrated that bee products were used to promote children’s health. The current research also showed that families use bee products for their children for both health protection and different purposes. The present study showed that the bee products consumed by families for their children were mostly honey and propolis. They generally mixed the bee products with the plant-based ingredients such as onion, ginger, and thyme. More than 50% of parents reported that bee products could have better effects when they were used with medicine. It was found that the use of bee products among pediatric pulmonology patients in South-East Serbia was widespread. Almost all interviewed parents reported a need to learn about bee products, their safe usage, and effects on children [34]. In the study conducted by Đorđević et al. [35], the attitudes and habits of Serbian preschool and school children regarding the consumption of meat products, milk and milk products, eggs and egg products, and honey and bee products were examined. The survey was conducted on 227 children, and the groups were divided according to the children’s age. It was found that preschool children consumed honey 14.99% more often than schoolchildren (7–11 years old), and 14.49% more often than did schoolchildren in grades 12–15. This study focused on children’s habits, and it did not include any parents’ opinions about the bee products.

In addition, the present study found that families prioritize bee products for their natural and additive-free qualities. Since these products are generally consumed for health purposes, they mustn’t contain chemical additives. Additives may reduce the nutritional value of products and cause allergic reactions or long-term health problems. Since young children may be more sensitive, parents might consider natural and additive-free bee products to be important criteria. In addition, the current study revealed that parents primarily purchase bee products from trusted/familiar producers, village markets, and pharmacies. In a study conducted by Živanović et al. [34], it was found that the products were generally purchased from pharmacies or directly from beekeepers. These findings have demonstrated that both trust and accessibility are effective in the supply of bee products.

The limitations of the study are listed below.

1. The participants of the study were limited to parents who have a child/children between the ages of 4–6 and live in the city center.
2. The parents who make up the study group were not kept equal in terms of being a mother or a father.
3. Since a purposeful sampling method was used in the study, although in-depth data on the subject were obtained, the findings could not be directly generalized to large masses due to the limitations of the sample.
4. The fact that the answers given by the parents to the interview questions were based on self-reports.

## 5. Conclusions

This study deeply explored the attitudes of parents about bee products by employing a qualitative study with semi-structured interviews with 40 parents of children aged 4–6 years. As the literature demonstrated that bee products have many positive effects on health, the obtained results also showed that the opinions of families were also important in the use of bee products on children. As a result of this study, it was found that families prefer natural products to protect their children’s health. It was seen that honey was most commonly used among bee products. It was determined that families are aware that the products are primarily natural and additive-free in bee products. It was clear that families hesitate to use products such as royal jelly and propolis because they may cause early puberty. This situation highlights the need for families to be better informed about the use of bee products, and further research should be conducted on the subject.

## Figures and Tables

**Table 1:** Demographic characteristics of the participants.

Categories		N	Categories		N
Gender	Female	26	Employment Status	Working	25
	Male	14		Not working	15
Age	20–29 years	2	Family structure	Nuclear family	34
	30–39 years	25		Extended family	4
	40–49 years	8		Single parent	2
Educational attainment	Primary and secondary school	1	Total number of children in the family	Only child	16
	High school	7		Two children	18
	Undergraduate degree	25		Three children	6
	Postgraduate	11	Total monthly income of your family	40,000 TL–60,000 TL	12
				More than 60,000 TL	28

## Data Availability

The original contributions presented in the study are included in the article; further inquiries can be directed to the corresponding author.
